# Objective sampling design in a highly heterogeneous landscape - characterizing environmental determinants of malaria vector distribution in French Guiana, in the Amazonian region

**DOI:** 10.1186/1472-6785-13-45

**Published:** 2013-12-01

**Authors:** Emmanuel Roux, Pascal Gaborit, Christine A Romaña, Romain Girod, Nadine Dessay, Isabelle Dusfour

**Affiliations:** 1 ESPACE-DEV, UMR228 IRD/UM2/UR/UAG, Institut de Recherche pour le Développement, Maison de la Télédétection, 500 rue Jean-François Breton, 34093 Montpellier Cedex 5, France; 2 Institut Pasteur de la Guyane, Unité d’Entomologie Médicale, 23 Avenue Pasteur, B.P. 6010, 97306 Cayenne Cedex, French Guiana; 3 Université Paris Descartes/PRES Sorbonne Paris-Cité, 19 rue de Dantzig, 75015 Paris, France

## Abstract

**Background:**

Sampling design is a key issue when establishing species inventories and characterizing habitats within highly heterogeneous landscapes. Sampling efforts in such environments may be constrained and many field studies only rely on subjective and/or qualitative approaches to design collection strategy. The region of Cacao, in French Guiana, provides an excellent study site to understand the presence and abundance of *Anopheles* mosquitoes, their species dynamics and the transmission risk of malaria across various environments. We propose an objective methodology to define a stratified sampling design. Following thorough environmental characterization, a factorial analysis of mixed groups allows the data to be reduced and non-collinear principal components to be identified while balancing the influences of the different environmental factors. Such components defined new variables which could then be used in a robust *k*-means clustering procedure. Then, we identified five clusters that corresponded to our sampling strata and selected sampling sites in each stratum.

**Results:**

We validated our method by comparing the species overlap of entomological collections from selected sites and the environmental similarities of the same sites. The Morisita index was significantly correlated (Pearson linear correlation) with environmental similarity based on i) the balanced environmental variable groups considered jointly (p = 0.001) and ii) land cover/use (p-value << 0.001). The Jaccard index was significantly correlated with land cover/use-based environmental similarity (p-value = 0.001).

**Conclusions:**

The results validate our sampling approach. Land cover/use maps (based on high spatial resolution satellite images) were shown to be particularly useful when studying the presence, density and diversity of *Anopheles* mosquitoes at local scales and in very heterogeneous landscapes.

## Background

Studies aiming to describe, understand and control vector-borne diseases are often based on field and/or remotely sensed data and on the use of Geographic Information System (GIS) tools [1-3]. Such methods aim to understand and predict target population features (such as vector occurrence or disease incidence) in space and time, and in relation to environmental factors. Population features are recorded by fieldwork or systematic monitoring. However, target populations are often too large, and the knowledge of their spatial and temporal distributions insufficient, to allow their exhaustive observation and characterization. When designing fieldwork sampling plans, researchers need to take into account financial, logistic, labor and ethical constraints in addition to the initial study objectives. To our knowledge, very few studies propose, at an early phase of research work, objective sampling strategies that are consistent with both study goals and constraints. When sampling strategy is based on environmental characteristics, studies may need to consider how best to find a manageable number of sites that are different but representative of the full environmental diversity [4]. Alternatively, they may need to consider how to find a manageable number of samples that share similar characteristics with a given target situation or population [5,6].

In each case, it is important to define consistent distances, dissimilarity or similarity indexes between all possible experimental situations in order to design an appropriate sampling strategy; this methodological issue is central to sampling stratification.

Hirzel and Guisan [7] compared four sampling strategies in a study of single species habitat suitability: regular, random, equal random-stratified and proportional random-stratified sampling. Although the authors used a simulated habitat suitability map based on eleven environmental variables, only the four most heavily weighted variables (*Forest frequency*, *Elevation*, *Aspect* and *Distance to towns*) were used to define the strata. This is quite realistic as, in practice, the environmental variables chosen for sampling design are often only a sub-set of the ones used, in a second stage, for studying the relationships between the environment and the field sampling results. The first stage ideally requires information on the entire study area (which can be costly, logistically difficult or even impossible to collate) whereas the latter one only concerns a limited number of sites and may cover relatively small areas. In their paper, Hirzel and Guisan defined 256 strata by combining four equal-range intervals of each of the four chosen environmental variables. The authors conclude that equal random-stratified and regular strategies provide better results. Among other factors improving sampling efficiency, they identified larger sample size and the use of environmental information. However, because simulated data was used, the environmental variables that better characterized species habitats were known and chosen to first define the strata. In practice, without background knowledge on species habitats or in the context of biodiversity assessments, where species can have a large range of habitats, environmental variable selection may not be obvious.

Keating et al. [8] proposed a proportional random-stratified sampling method to study the relationships between urban and peri-urban characteristics and African malaria vectors. Five strata were defined on a two-criterion basis: extent of urban planning and degree of drainage. Strata were defined by examining objective information provided by district development plans, town maps, GIS base maps, ground truthing, topographic features, house distribution, presence of engineered drainage systems, types and patterns of roads, and sources of community water. However, no detail is given on how information was exploited to define the strata, which seems to be the result of expert interpretation.

In Pope et al. [9], sites for malaria vector habitat characterization were chosen by defining two marsh classes: impacted marshes (with sugar cane fields adjacent to the marsh) and unimpacted marshes (with forest or scrub surrounding the marsh). Four sampling periods were defined according to the typical annual precipitation cycle, allowing the habitat to also be characterized by time. In this case, spatial stratification is defined by only one categorical variable: the presence of sugar cane fields adjacent to the marsh. In contrast, for more exploratory studies where species and habitats are not known, ideally many more environmental variables would have to be taken into account.

Danz et al. [4] proposed a sampling design to elaborate indicators of anthropogenic impacts on the environment. They used a stratification procedure to ensure that samples were distributed across important anthropogenic stress gradients. Seven categories corresponding to different environmental factors (such as agriculture and atmospheric deposition) were used to group the 207 environmental variables. The effects of these categories were balanced before applying a non-supervised clustering method to define the strata for the selection of final samples. We chose to apply a similar approach to assess the presence, abundance and distribution of adult anopheline mosquitoes in French Guiana and to characterize the environmental determinants of malaria transmission. We hypothesize that the environment contributes, even at a very local scale, to structure of adult anopheline populations. However, our method offers both a more unified theoretical framework and wider applicability.

Malaria is a major public health issue in French Guiana where about 3,450 acute cases have been recorded each year during the last decade [10]. A drastic drop in cases has occurred the last two years with only 1,400 (on average) being recorded between 2010 and 2011. Most transmission occurs inland, along the rivers, whereas the coastal areas inhabited by 75% of the population are almost free of autochthonous cases [11]. However, entomological surveys show that *Anopheles darlingi* and several other potential malaria vectors are present in the littoral zone [12,13]. Human population flow between the inland and littoral areas, as well as across country borders, is also increasing. As a consequence, risk of malaria epidemics in littoral areas has become higher, and episodes of malaria outbreak have already been experienced by the coastal population at some focal points. This is true of the village of Cacao, where 43% of the inhabitants have experienced malaria (mainly *Plasmodium vivax*) between 2002 and 2007 [14]. *Anopheles darlingi* is considered as the primary vector in the region. However, preliminary studies have highlighted high diversity of adult anopheline mosquitoes in the village and its surrounding area, suggesting there may be other potential *Anopheles* species acting as natural vectors in French Guiana [15]. The littoral area, particularly the region of Cacao, therefore provides an excellent study area for understanding anopheline species dynamics, species distribution and malaria transmission risks across diverse environments. Therefore, we planned additional mosquito collections to study the *Anopheles* species present in the village area and further explore the results found in the preliminary studies.

Given the constraints imposed on entomological surveys in remote areas, the study area specificities and the lack of adapted sampling design method in the literature, there was a need for methodological development to objectively design the sampling strategy. We proposed an original and generic methodology in order to define an appropriate sampling strategy with two main objectives: i) to maximize the chance to observe the entire species diversity and ii) to characterize the species habitats within a highly constrained framework in term of number of sampling sites.

We designed an objective stratified sampling strategy based on an intensive environmental characterization using remote sensing data, Geographic Information System (GIS) tools, data analysis and clustering. Then we evaluated this strategy according to adult mosquitoes collected.

## Methods

### Study area

**Figure 1:**
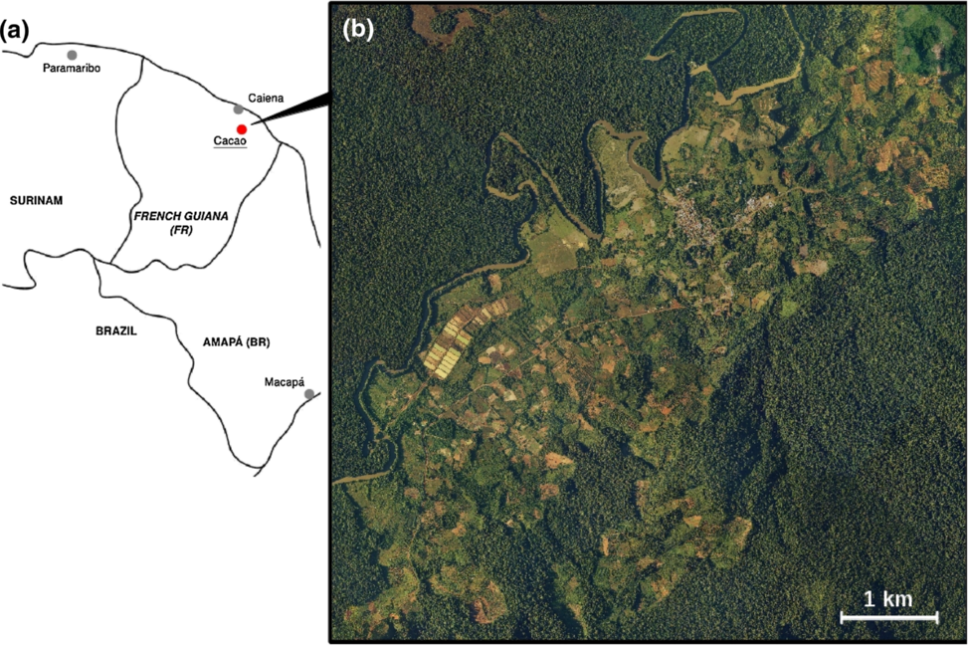
**Study area.** **a)** Localization of French Guiana and the region of Cacao; **b)** Aerial photographs of the Cacao village, the cultivated area and the surrounding dense forest, acquired in 2006 (BD Ortho product from IGN, the French National Institute of Geographic and Forest Information). The wide and sinuous river at the northwest is the Comté River.

French Guiana is a French overseas region located in South America. The territory is separated from Brazil and Suriname by the Oyapock and Maroni rivers, respectively, and is largely covered by the Amazon forest (about 94% of its area). The village of Cacao was established in 1977 in a forested area 75 km southeast of Cayenne, the main town of French Guiana (see Figure 1a). The total population is about 839 inhabitants [14].

Cacao is mainly inhabited by Hmong people who fled refugee camps in Laos during the 1970s. Intensive agriculture was developed in the area and now represents a large part of the production of vegetables and fruits in French Guiana. Farming, aquaculture and tourism complement the range of village activities. These intensive and various activities result in a very fragmented and heterogeneous land cover/use (see Figure 1b).

The village is surrounded by Amazonian rainforest and has a hot (mean temperature = 27°C) and humid (mean relative humidity = 80%) climate all year round. As in all of French Guiana, there are four distinguishable seasons: a long rainy season (mid-April - mid-July), a long dry season (mid-July - November), a short rainy season (December - January) and a short dry season (February - mid-April). Cacao is situated in the most rainy part of French Guiana, with about 3,800 mm of cumulative rainfall on average each year.

### Sampling design

#### Sampling area

Two criteria were used to define the study area. Firstly, the potential sampling area had to be accessible for the collection of adult mosquitoes: it laid on the right bank of the Comté River (i.e. mainly on the east bank of the river), less than 250 m from roads or trails and did not include water surfaces. Secondly, the village (defined by dense building zones and manually digitized) was to be considered as a stratum. This was *a priori* considered given the desire to establish a mosquito collection site in the most densely populated area when evaluating malaria transmission risk. The village was therefore excluded from the objective stratification procedure described below.

#### Virtual sampling sites

Five hundred sites were defined by uniformly random sampling. These are referred to as *virtual* sites as they formed part of the stratification procedure but were not the final sampling sites.

#### Environmental data

##### 

**Land cover/land use** Only one scene of the SPOT-5 satellite is sufficient to cover the study area. However, due to the omnipresence, in this region, of clouds and cloud shadows that generate missing data, five SPOT-5 satellite images covering the study area, with four color channels (red, green, near infrared and middle infrared) and at 10-meter spatial resolution, were selected. They were selected according to having been taken under the best cloud conditions (low cover), at dates concurrent with the beginning of the mosquito collection and with small time intervals between acquisitions (to minimize the risk of change in land cover/use during this time). Selected images were acquired during the long dry season, on August 1^st^ and 22^th^, September 1^st^ and 11^th^, and October 13^th^ 2009. This was the only period of suitably low cloud cover. Images were provided by the SEAS-Guyane project ( http://www.seas-guyane.org).

Five land cover/use maps were produced, using a semi-supervised classification also applied in [16,17]. This classification procedure combined a *k*-means clustering of the image pixels and the intervention of an operator, the first author of this paper, who was guided by field knowledge, expertise in photo-interpretation and collaboration with entomologists. More precisely, a *k*-means clustering (with *k* = 50) was first applied to the image pixels, and eleven pre-labeled land cover/use classes were manually defined by the operator, by merging initial clusters. A set of classification rules was then applied to remove confusions in the initial pre-labeled classes. Such rules took into account patch sizes, distributions and relations. Such “structural” knowledge appeared to be more robust than the ones provided by the pixel radiometric values. Eventually, a final correction was manually performed to remove the remaining errors. Aerial photographs acquired in 2006 by the IGN, the French National Institute of Geographic and Forest Information (BD-Ortho®;product), with a spatial resolution of 50 cm, were visually interpreted for the labeling of land cover/use classes and used for qualitative validation of the classification. Then, a synthetic land cover/use map was produced by combining the five initial maps, in order to fill in the missing data due to clouds and cloud shadows as much as possible. Ten land cover/use classes were identified: dense forest, secondary/degraded forest (including “old” fallows – approximately 6-meters high – with heterogeneous and dense vegetation), mono-specific and homogeneous vegetation (bamboos, cecropias, more recent fallows), dense low vegetation with shrubs, dense low vegetation, scattered herbaceous vegetation, dry savannah (during dry season), bare soil, water, and no data (due to clouds and cloud shadows). These classes correspond to both a gradient of vegetation density and height. Such classification procedure was implemented with the free and open software GRASS GIS [18] and with the free software environment for statistical computing R [19]. The detailed algorithm, in pseudo-code, is presented in Additional file 1.

##### Complementary geographic information layers

Table 1 lists the complementary geographic information layers used in our study, their sources, the methods used to create them and the environmental variables derived from them. These layers characterize human activity/planning (asphalt roads, trails, buildings, greenhouses, aquaculture basins and gold mining sites), hydrology (permanent and temporary rivers, natural water surfaces and floodplains) and topography (altitude, slope and orientation).

**Table 1:** Complementary geographic information layers, data sources and attributes extracted for landscape characterization

**Geographic information layer**	**Information source**	**Method for layer creation**	**Remarks**	**Attributes extracted for virtual sites**	**Variable group**
Buildings				Minimum distance to, Number^c^	Anthropization
Asphalt roads, trails	Air photographs^a^	Manual digitizing (digitizing scale ≈ 1/5400)	Roads/trails accessible with a four-wheel drive vehicle	Minimum distance to (m), Length (m)^c^	
Greenhouses				Minimum distance to, Number^c^	
Anthropogenic basins	Air photographs^a^ and BD-Carto®;^b^		Basins may or may not be permanently flooded	Minimum distance to (m), Surface proportion^c^	
Gold mining sites	Air photographs^a^ and SPOT5 satellite images	Manual digitizing		Minimum distance to (m)	
Permanent rivers (excluding the Comté River)	BD-Carto®;^b^			Minimum distance to (m), Length (m)^c^	Hydrology
Temporary rivers (excluding the Comté River)					
Banks of the Comté River	Land cover map	GIS^d^ computation from the land cover map			
Natural water surfaces	Air photographs^a^ and BD-Carto®;^b^		May or may not be permanently flooded	Minimum distance to (m), Surface proportion^c^	
Floodplains			Temporarily flooded		
Altitude				Altitude (m)	Topography
Aspect (positive angle, in degree, relative to a west-east line)	SRTM^e^ Digital Elevation Model	GIS^d^ computation from SRTM data		West-East orientation (cosine), South-North orientation (sinus)	
Slope				Slope (%)	

^a^Aerial photographs from BD-Ortho®; 2006 product (IGN, the French National Institute of Geographic and Forest Information).

^b^BD-Carto®; 1:25,000 scale map (IGN product, 2006).

^c^Attribute extracted within a 200 m radius buffer, centered on the virtual collection site.

^d^GIS: Geographic Information System. The fee and open software used is GRASS GIS [18].

^e^SRTM: Shuttle Radar Topography Mission (90 m spatial resolution).

#### Landscape characterization

For each virtual sampling site, the land cover/use map was used to calculate the following variables: i) the minimum distance to unfragmented forest (defined as an unbroken patch of primary forest surrounding the anthropized area), ii) the proportions of the land cover/use classes within a discoidal buffer of 200-meter radius, iii) the landscape division according to Jaeger [20] and iv) the minimum distances to each land cover/use class. For this last set of variables, patches less than 500 m^2^ were excluded because pixel-based image classifications may generate many isolated and non-significant patches and therefore misleading results. Attributes were also extracted from each complementary geographic information layer (see Table 1). In total, for each of the 500 virtual sites, 43 attributes were extracted from the different layers. Sites for which the 200 m radius buffer had 5% or more land cover/use information missing, were excluded, leading to 484 remaining sites for the study. The landscape characterization was performed with the free geographical information system GRASS GIS [18] and especially the toolbox for the quantitative analysis of landscape structure, *r.le.patch*[21].

#### Data coding

**Figure 2:**
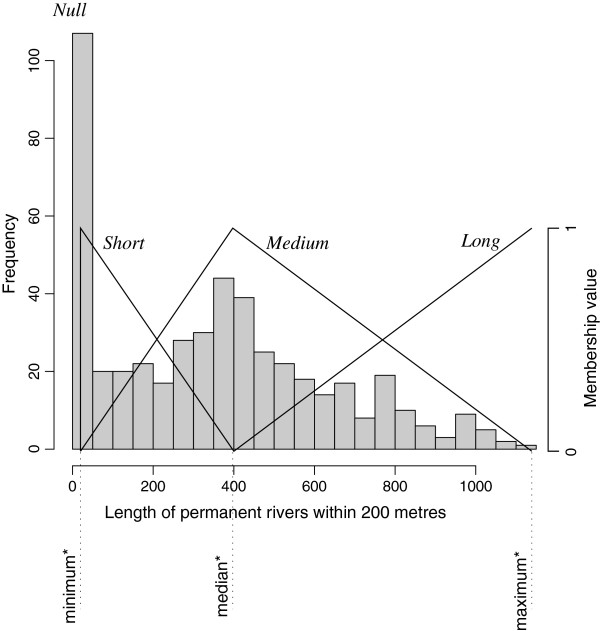
**Fuzzy data coding.** Example of fuzzy categorization of a real continuous variable. The asterisk indicates that the minimum, median and maximum values are computed on non-null values only.

Variables with a strong positively or negatively skewed distribution were subjected to square-root or square transformations, respectively. Some buffer-based landscape attributes exhibited a “threshold effect”: if the environmental feature of interest was present within the buffer the sites scored non-null values with a given distribution but, in some cases, a significant number of sites returned a null value as the feature did not exist within the buffer (see Figure 2). For such variables, we coded data by computing their membership values with fuzzy intervals, transforming real continuous variables into categorical ones with three categories (or “modalities”) [22-24]. Membership functions are triangular functions, as illustrated in Figure 2.

To be defined, such membership functions require only three values for each variable (see Figure 2). In our case, for each variable to be fuzzified, we chose the minimum, the median and the maximum values of the observed non-null values.

Two other variables had very few non-null values and were re-coded as binary presence/absence data: the proportion of natural water body surfaces within 200 m and the proportion of floodplain surface within 200 m.

The 43 environmental variables were assigned to homogeneous groups related to specific “environmental points of view” or “environmental factors”. One group, defined by all the variables derived from the land cover/use map (proportions, distances and landscape division), is referred to as the land cover/use group. The other variable groups (Table 1) were associated with topography, human activity/planning and hydrology.

#### Stratification (clustering)

We first applied a factorial analysis of mixed groups (FAMG) [25-27] on the environmental variables. The FAMG first performs separate PCAs on each variable group before a weighed PCA is applied to the entire variable set. The global PCA uses the inverse of the maximum eigenvalues found from each separated PCA, as the weights for each variable group. This method consequently balances the maximum inertia of each group in the overall analysis. This was done to avoid variable collinearity, identify the significant environmental information and to balance the influence of the variable groups. This variable group balancing was explored in our study as it was assumed that different environmental factors may have comparable weights in explaining the presence, density and distribution of the different *Anopheles* species in Cacao. Such an assumption seemed the more consistent in the framework of our application and is consistent with many practical situations. In fact, firstly we did not know, *a priori*, what were the effective relative impacts of the environmental factors and, secondly, we wanted to avoid that the relative influences resulted from an analysis bias due to difference in the number of variables in each group.

We then applied a *k*-means clustering procedure to the virtual site coordinates in the factorial space provided by the FAMG. For this, we considered the most informative FAMG axes which represented 80% of the cumulated data variance. We set the number of expected clusters to five, as the total number of sampling sites out of the village was fixed to ten according to our sampling capacity (see following paragraph for details), each cluster defining a sampling stratum. The sixth stratum was the Cacao village itself (the most urbanized zone of the study area).

#### Choice of the final mosquito collection sites

Based on the resulting clusters, twelve mosquito collection sites were chosen: one main and one secondary site per cluster. The choice of site also took the following field constraints into account: accessibility due to vegetation density, slope of the terrain, presence of water or fences, disturbances of farm work and inter-site distances (in order to limit the travel time between sites during mosquito collection sessions). The secondary site was allocated in case of non-accessibility of the main site at the time of collection and to investigate landscape context intra-variability, in terms of anopheline mosquito occurrence.

### Validation of the method

#### Collection of adult mosquitoes and identification of *Anopheles* species

The human landing technique was used. Female mosquitoes were collected using mouth aspirators when landing on collector forelegs. Collectors were members of IRD (French Research Institute for Development) and IPG (Pasteur Institute of French Guiana) teams and local volunteering residents to which the method and risks were fully explained. All collectors gave their free, express and informed consent for mosquito collection and were supervised during the captures by either an IPG or IRD staff member. Malaria prophylaxis with the combination of atovaquone and proguanil hydrochloride (Malarone®;) was also proposed and information on the medication were provided. Collectors who benefited from prophylaxis gave their free, express and informed consent.

The human landing technique was carried out for 432 man-hours at each location. Mosquitoes were collected during four sessions taking place from 5:00 to 7:00 am and from 6:00 to 10:00 pm. All anopheline mosquitoes were identified by morphological identification keys used in the area [28-31].

#### Similarity indexes

*Anopheles* species composition at the collection sites was assessed and compared. The Jaccard similarity index [32] (which considers only presence/absence data) and the Morisita index [33] (which also considers abundance data) were computed. To validate our approach, these similarity indexes were compared with environmental similarities. By considering the Euclidean distance, *d*, between two capture sites *i* and *j* within the environmental variable space provided by the FAMG, we defined the environmental similarity for each unique pair (*i*,*j*) of capture sites (with *i* ≠ *j*), as follows:

(1) $$\begin{array}{l} S_{e}{\left(i,j\right)}_{i\neq j}\\ =1-\frac{d(i,j)-{\text{min}}_{\{k,l\}\in C\;\text{and}\;k\neq l}(d(k,l))}{{\text{max}}_{\{k,l\}\in C\;\text{and}\;k\neq l}(d(k,l))-{\text{min}}_{\{k,l\}\in C\;\text{and}\;k\neq l}(d(k,l))} \end{array}$$

*C* being the set of all unique capture site pairs, the number of which is, for *N* sites, equal to $\frac{N\times (N-1)}{2}$.

FAMG is able to provide partial views of the statistical individuals (the virtual sites in our case) within the factorial space, i.e. their coordinates according to the different variable groups. This enabled definition of topographical, anthropogenic, hydrological and land cover/use similarities.

## Results

### Sampling stratification and characterization of the landscape contexts

**Figure 3:**
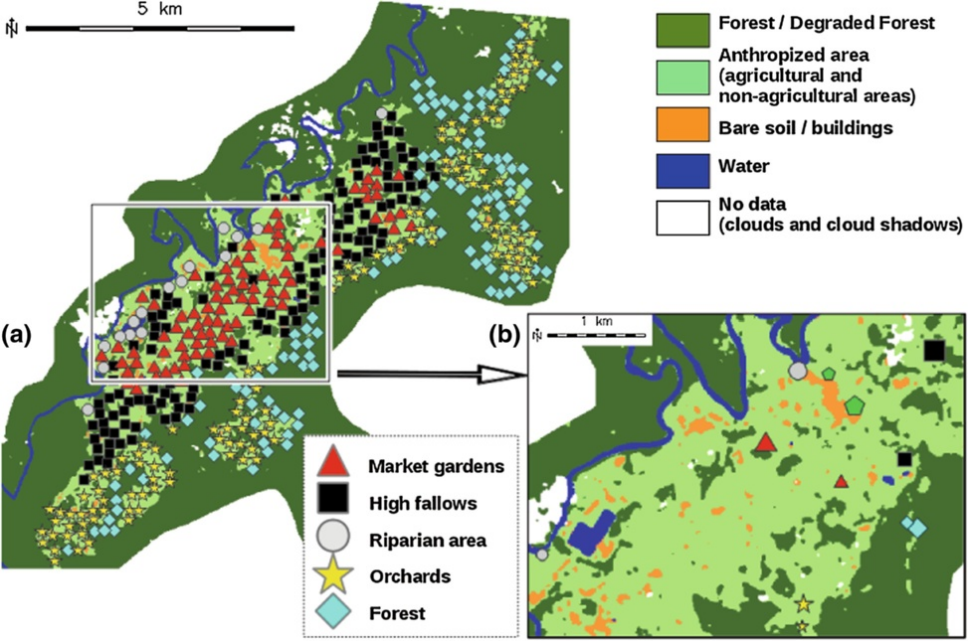
**Virtual, final collection sites and landscape contexts.** **a)** Simplified land cover/use map, virtual sites and their memberships to the five clusters (defining landscape contexts); **b)** Simplified land cover/use map, chosen “real” mosquito collection sites and their memberships to the five clusters. Large and small symbols correspond to main and secondary mosquito collection sites, respectively.

Figure 3a shows the five virtual site clusters in geographical space. Clusters define consistent geographical zones (described in Table 2) according to the geographic information layers, the field observations and the FAMG results.

**Table 2:** Description of the five clusters

**Symbol and color (see Figures**3**and**4**)**	**Characteristics in the geographical space (Figure**3**)**	**Characteristics in the environmental variable space (Figure**4**)**	**Name of thelandscape context**
Light gray disks	Sites located very near the Comté river	Far from gold mining sites and unfragmented forest; close to floodplains, the Comté river and water (from remote sensing); low altitude and slope; long or medium lengths of Comté river banks within 200 m	**Riparian area**
Red triangles	Sites located on a plain, corresponding to a zone devoted to mixed vegetable gardening	Far from gold mining sites and unfragmented forest; close to buildings, water, basins and greenhouses; low proportion of forest; medium number of building; medium and high number of greenhouses	**Market gardens**
Yellow stars	Sites located on hills within a zone devoted to fruit culture	High altitude and slope, far from water, the Comté river, flood plains, greenhouses and basins; within 200 m: no buildings, greenhouses or basin; short lengths of roads; length of the Comté river null; high proportion of forest	**Orchards**
Blue diamonds	Isolated sites in non or slightly degraded forest	Shares the majority of *Orchards* landscape context features, but exhibits higher distances to buildings, shorter lengths of road within 200 m and lower distances to unfragmented forest	**Forest**
Black squares	Sites situated between the market gardens and the orchards	Difficult to characterize by interpreting the FAMG. Field observations tend to associate such a cluster with i) the presence of very degraded forest patches which are difficult to exploit because of swamps, or ii) numerous patches of fallowed land (4 years and older), corresponding to a stage of the crop rotation	**High fallows**

**Figure 4:**
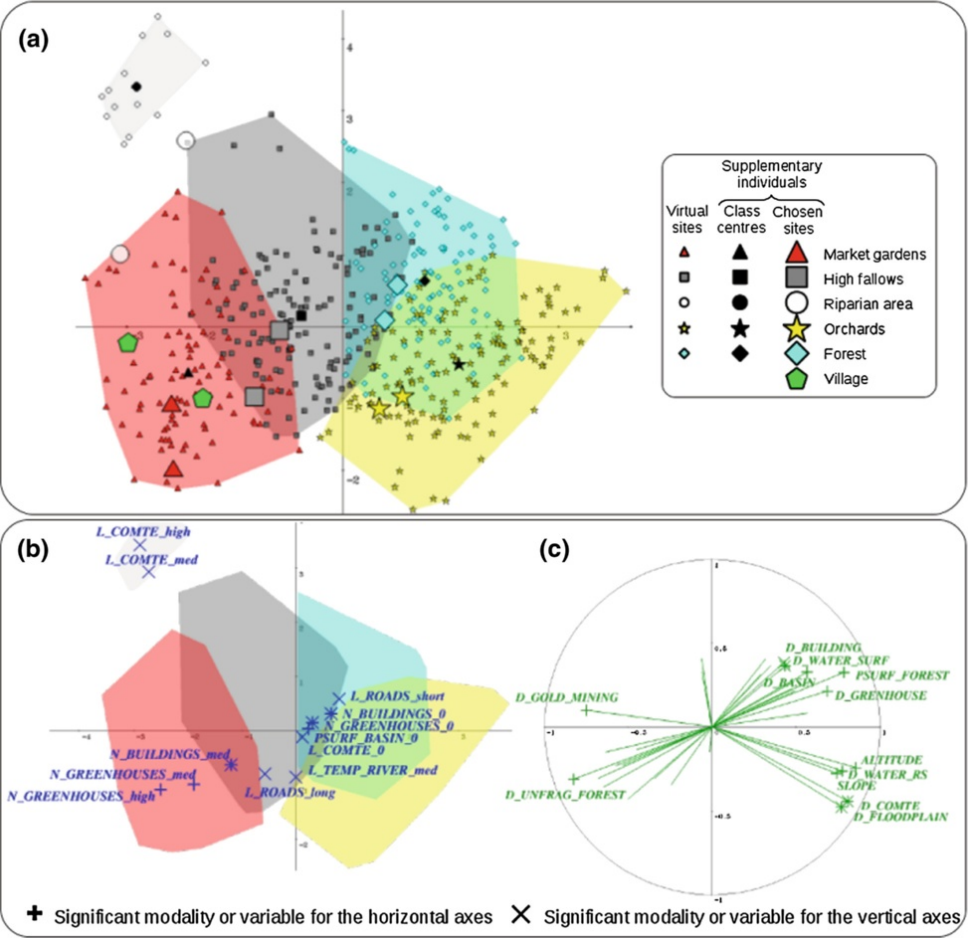
**Results of the factorial analysis of mixed groups.** **a)** Virtual and chosen mosquito collection sites on the first factorial plane (factorial axes 1 and 2) of the FAMG; **b)** significant modalities and **c)** quantitative variables for the first factorial plane of the FAMG. The colored polygons in sub-figures a) and b) delimit the five clusters provided by the *k*-means procedure.

Figure 4 illustrates the virtual sampling sites, the modalities of the categorical variables and the quantitative variables projected onto the first factorial plane of the FAMG (summarizing 27.1% of the total data variance). We show only variables and modalities that significantly contribute to, and that are well represented on the axes: we computed the total contributions of categorical variables by summing the contributions of their modalities, and then ranked all the variables in descending order of their contributions; a variable was then considered as contributing significantly to the axis if it fell within the cumulative contribution of up to 75%; a quantitative variable or a categorical variable modality was well represented on the factorial axis if its quality of representation (*cos*^2^) on the axis was superior to 0.1.

A cluster at the top left of Figure 4a appears to be atypical. The four remaining clusters are distributed along the first factorial axis. The detailed interpretation of the FAMG is presented in Table 2. Moreover, by considering the cluster characteristics in both geographical and environmental variable spaces, it is possible to assign each cluster to a homogeneous landscape context (see Table 2), that is related to particular geographical locations and to topographical, hydrological, anthropogenic and land cover/use specificities (see Table 2).

### Choice of final collection sites

Based on the FAMG results and field observations, twelve capture sites (one main site and one secondary site per landscape context) were chosen (see Figure 3b).

### Mosquito collection and identification

Mosquito collections were performed at the main and the secondary sites for the landscape contexts *Village*, *Orchards* and *Market gardens*. Only the main collection site was considered for *High fallows*, *Forest* and *Riparian area* contexts. Consequently, nine collection sites were considered, providing 36 unique site pairs for the computation of similarity indexes.

A total of 829 females from ten *Anopheles* species were caught during the collections. Among them, the major malaria vector in Latin America (*Anopheles darlingi*) and other secondary or occasional vectors in the Amazonian region (*An. braziliensis*, *An. intermedius*, *An. nuneztovari*, *An. oswaldoi* and *An. triannulatus*), were found. Table 3 lists the ten collected species and details the number of mosquitoes for each species as a function of the landscape contexts defined for sampling stratification.

**Table 3:** **Number and species richness of *Anopheles * mosquitoes, as a function of the landscape context**

**Species**	**Riparian area**	**Forest**	**Orchards**	**High fallows**	**Market gardens**	**Village**
*An. braziliensis*	2	-	-	-	1	2
*An. darlingi*	**81**	-	6	10	**78**	**97**
*An. ininii*	-	1	-	1	-	-
*An. intermedius*	4	-	-	1	5	-
*An. mediopunctatus*	1	3	1	3	1	-
*An. nuneztovari*	14	-	4	15	14	1
*An. oswaldoi*	-	1	-	-	-	-
*An. peryassui*	1	-	-	-	-	-
*An. strodei*	1	-	-	-	1	-
*An. triannulatus*	25	**30**	**16**	**404**	4	-
**Total**	**129**	**35**	**27**	**434**	**104**	**100**
**Species number**	**8**	**4**	**4**	**6**	**7**	**3**

### Sampling design evaluation

**Figure 5:**
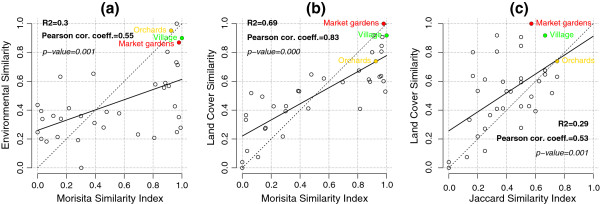
**Environmental similarities** ***vs.* Morisita or Jaccard index for significant Pearson correlation coefficients.** **a)** Environmental similarity for all unique pairs of sites, by considering all the environmental factors jointly and as a function of the Morisita index; **b)** Land cover/use similarity as a function of the Morisita index; **c)** Land cover/use similarity as a function of the Jaccard index. The dotted line corresponds to the ideal case where similarities are equal. The continuous line corresponds to the linear regression result; the associated *R*^2^, Pearson correlation coefficient and *p*-value are shown at the top left. Points corresponding to the same landscape context are labelled with the name of context.

The Figure 5 presents the results of the similarity indexes for significant correlations (in term of Pearson correlation coefficient) between environmental similarities, Morisita (Figures 5a and 5b) and Jaccard indexes (Figure 5c).

The Morisita similarity index appears significantly correlated with the environmental similarity by taking into account all the environmental factors jointly (Pearson correlation coefficient, *ρ* = 0.55, p-value = 0.001, Figure 5a) and strongly correlated with the land cover/use similarity (*ρ* = 0.83, p-value << 0.001, Figure 5b). However, similarities computed for topographical, anthropogenic or hydrological factors are not significantly correlated with the Morisita index.

The two sites (main and secondary) belonging to the same landscape context are similar in terms of Morisita and environmental similarities by taking into account the environmental factors jointly and by considering the hydrological and the land cover/use factors individually (with similarities above 0.7) (see Figures 5a and 5b). However, the similarities between main and secondary sites by considering the topographical and anthropogenic factors are low: the two sites belonging to the *Village* exhibiting topographical and anthropogenic similarities equal to, respectively, 0.47 and 0.55, and the two sites belonging to the *Market gardens* landscape context exhibiting an anthropogenic similarity of about 0.4 only.

The environmental similarity based on land cover/use is the only one significantly linked to the Jaccard similarity (linear correlation and simple linear regression, *ρ* = 0.53, p-value = 0.001; see Figure 5c). Moreover, similarities according to the Jaccard index, for sites within the same landscape context, are lower than with the Morisita measure (Figure 5c).

## Discussion

We designed a sampling strategy for adult anopheline mosquito inventory and habitat characterization, based on environmental stratification. Stratification was provided by an *a priori* balancing of information brought by different groups of environmental variables: topographical, anthopogenic, hydrological and land cover/use. The approach is comparable to that of Danz et al. [4] in which one Principal Component Analysis (PCA) was applied on each category (called “group” in our case) of quantitative environmental variables. The total variance of the categories was balanced in order to equalize the variable categories in the clustering process and the rescaled principal component scores provided by the separated PCAs were used directly for clustering. As a global PCA was not performed on all the rescaled categories, variables were uncorrelated within each category but may have been correlated from a category to another. Here, we applied a factorial analysis of mixed groups (FAMG). This method balances the maximum inertia of each group in the overall analysis and provides uncorrelated principal component scores as inputs for *k*-means clustering. An FAMG also enables both categorical and quantitative variables to be considered in the analysis. This allows i) initial quantitative variables to be recoded as categorical ones if they exhibit atypical distributions, ii) non-linear relationship between variables to be considered and iii) categorical variables provided by surveys to be taken into account. We therefore suggest that our approach may offer both a more unified theoretical framework and a wider applicability than in Danz et al. [4].

In our study, some landscape descriptors were extracted from a 200 meters radius buffer. This radius was chosen as a compromise between a relevant landscape characterization according to the satellite image spatial resolution (10 meters) and the overlap between neighbour buffers (i.e. information redundancy and spatial auto-correlation). Given the number of *Anopheles* species involved, the lack of knowledge on their ethology and the lack of pre-existing field data, we had not enough information to objectively choose a radius as it was the case in Stefani et al. [17], where a model selection approach was applied (by using linear regression models and the Akaike criterion) to objectively define the best radius in their specific application framework. By using the collected data in Cacao, we should consider such an objective radius selection in the future, and an *a posteriori* discussion of the choice (200 m) made for the present study should be conducted.

The results validate our approach in that the similarity measure we defined (considering the balanced variable groups taken jointly) is significantly correlated with the Morisita similarity measure. Our sampling design appears appropriate and relevant for i) optimizing collections for species inventory and ii) studying the environmental determinants of the insect presence and density.

We were also able to consider the different environmental variable groups separately. Results show that the group derived from the land cover/use map was the only one that significantly correlated with both the Morisita and Jaccard indexes. This questions the initial hypothesis that environmental factors play equal roles in species occurrence. As a consequence, the strata (the different landscape contexts defined), can also be questioned. However, in practice, the same stratification procedure performed on the principal component scores provided by a mixed data analysis (without balancing the environmental factors) [26] gave comparable clusters in terms of spatial and environmental descriptions, and did not modify the membership of the final mosquito collection sites to the landscape contexts (data not shown).

We show that remote sensing data, through the characterization of land cover/use, is most likely to characterize the diversity and abundance of *Anopheles* species in Cacao. This could be applicable to other areas and application domains. Our results indirectly but objectively validate the information provided by the land cover/use map and justifies the use of high resolution remote sensing for the study of the adult *Anopheles* habitats at a very local scale. It also suggests that spatial interpolation and extrapolation of results are possible, based on the quasi continuous and complete land cover/use information derived from the imagery.

At this stage, it seems legitimate to wonder whether the simple geographical distances could explain the ecological diversity as well or better than the environmental distances within the FAMG factorial space. Using Pearson correlations we found no significant relationship between geographical similarity (computed by means of Equation (1) with *d* as the geographical Euclidean distance) and either the Morisita (p = 0.068) or Jaccard (p = 0.985) indexes.

The landscape contexts seem distributed perpendicularly to the Comté river (see Figure 3a). This direction corresponds to an altitude gradient (the *Altitude* variable being correlated with the first factorial axis of the FAMG; correlation coefficient = 0.86). Therefore, we could also wonder if the inter-site Euclidean distances in the geographical space, along this direction only, could significantly explain the ecological diversity. Consequently, we computed such distances and compared the derived similarities (obtained with Equation (1)) with the Morisita and Jaccard indexes. A significant correlation (*ρ* = 0.4 and p-value = 0.016) was found for the Morisita index only. However, this relationship remains less significant than with the balanced environmental characterization or that derived from the land cover/use characterization. This supports objective environmental characterization and, in particular, the use of land cover/use cartography.

## Conclusion

This work proposes a sampling design methodology intended for highly heterogeneous landscapes where sampling efforts for studying species occurrence and habitat are constrained. It is applied specifically to anopheline mosquitoes in the region of Cacao, French Guiana. However, the methodology is applicable to other contexts. We evaluate the results of our procedure with field collections. Results validate our approach and identify the value of land cover/use maps for the study of the presence and density of *Anopheles* species at local scales and in very heterogeneous landscapes. However, we also demonstrated that different environmental factors can influence species presence and density in different ways. Ideally, different combinations of *a priori* influencing factors should be considered to identify robust clusters and ensure non-biased field sampling.

This type of analysis improves the hazard assessment within the context of malaria transmission risk analysis in French Guiana, and more generally in the Amazonian region. As the proposed approach could also permit categorical survey variables to be considered, it allows exploration of the socio-economic, behavioral and perceptive dimensions associated with the risk of malaria transmission.

## Authors’ contributions

ER participated in research design, land cover/use map production, data collection, analysis and interpretation, and preparation of the manuscript. PG was involved in the field work planning and realization, the mosquito identification and revision of the manuscript. CAR, RG and ND participated in research design and revision of the manuscript. ID was the scientific coordinator of the project “Bioecology of the vectors of malaria in Cacao, French Guiana: towards assessing the exposure risk and improving the vector control”, within which this study was performed. She participated in research design, data collection, analysis and interpretation, and revision of the manuscript. All authors read and approved the final manuscript.

## Supplementary Material

Additional file 1 Commented algorithm, in pseudo-code, used to obtain the land cover/use map from SPOT5 images. Click here for file
