# Supplementary material for: Objective sampling design in a highly heterogeneous landscape - characterizing environmental determinants of malaria vector distribution in French Guiana, in the Amazonian region
Source: BMC Ecol. 2013 Dec 1;13:45. doi: 10.1186/1472-6785-13-45 (PMC4219608; doi:10.1186/1472-6785-13-45)
Supplement: Additional file 1 — Commented algorithm, in pseudo-code, used to obtain the land cover/use map from SPOT5 images. [file 1472-6785-13-45-S1.pdf]

Additional file 1/1

Objective sampling design in a highly heterogeneous landscape - characterizing environmental determinants of malaria vector distribution in French Guiana, in the Amazonian region

Algorithm 1 : Commented algorithm, in pseudo-code, used to obtain the land cover/use map from SPOT5 images

\# **Notations**
\# C_i: classe i
\# p_k: patch k (patch = set of contiguous pixels belonging to the same class)
\# $|p_k|$: number of pixels of patch k
\# $|p_k \cap p_l|$: overlap, in number of pixels, of patches k and l
\# class(p_k): returns the class to which the patch p_k belongs
\# class(p_k) $\leftarrow C_i$: the class of the patch p_k is set to C_i
\# p_k^L: k^{th} patch associated with layer L
\# $P_{C_i} = \{p_k | class(p_k) = C_i\}$: set of patches associated with the class C_i

for $i = 1$ to 5 **do**
  Perform a k-means clustering of the pixels of the image i (four bands), with $k = 50$

  \# Create initial land cover/use map, Map_i, with pre-labelled classes that include some land cover type confusions (that can not be removed at this step) (expert-based):
  Merge initial clusters to define pre-labelled classes (with confusion): C_1 = *Cloud OR Bare soil OR Building OR Unknown*; C_2 = *Could shadow OR Water OR Unknown*; C_3 = *Bare soil OR Building OR Unknown*; C_4 = *Water*; C_5 = *Forest*; C_6 = *Secondary growth OR Degraded forest OR Forest*; C_7 = *Vegetation 1 (anthropogenic)*; C_8 = *Vegetation 2 (anthropogenic)*; C_9 = *Vegetation 3 (anthropogenic)*; C_{10} = *Vegetation 4 (anthropogenic)*; C_{11} = *Vegetation 5 (anthropogenic)*

  \# Detect clouds and cloud shadows:
  Create layer L with P_{C_1} and layer L' with P_{C_2}
  Find translation t that maximizes $\sum_{(p_k^L, p_l^{L'}) \in L \times L'} \left(\mathrm{t}(p_l^{L'}) \cap p_k^L\right)$
  for all $(p_k^L, p_l^{L'}) \in L \times L'$ **do**
    if $\frac{|p_k^L \cap \mathrm{t}(p_l^{L'})|}{|p_k^L|} > \alpha$ **then** \# α is found with a trial-and-error approach
      class(p_k^L) $\leftarrow C_{11}$ \# C_{11} = *Cloud*
      class($p_l^{L'}$) $\leftarrow C_{21}$ \# C_{21} = *Cloud shadow*
    else
      class(p_k^L) $\leftarrow C_3$ \# C_3 = *Bare soil OR Building OR Unknown*
      class($p_l^{L'}$) $\leftarrow C_{22}$ \# C_{22} = *Water OR Unknown*
    end if
  end for
  Update Map_i

Reclass small isolated patches labeled $C_3 =$ *Bare soil OR Building OR Unknown* that are far from water[(1)] as $C_{32} =$ *Unknown*:

Create layer L with P_{C_3} and layer L' with $P_{C_3 \cup C_4}$
Create layer L'_{closed} by applying to L' a closing morphological filter with a 5-pixel radius disk as structuring element
Create layer $L'_{closed\ filtered}$ by removing small patches (< 0.1 ha) from L'_{closed}
for all $(p_k^L, p_l^{L'_{closed\ filtered}}) \in L \times L'_{closed\ filtered}$ **do**
  if $|p_k^L \cap p_l^{L'_{closed\ filtered}}| > 0$ **then** # In fact, in this case, $|p_k^L \cap p_l^{L'_{closed\ filtered}}| = |p_k^L|$
    class$(p_k^L) \leftarrow C_{31}$ # $C_{31} =$ *Bare soil OR Building*
  else
    class$(p_k^L) \leftarrow C_{32}$ # $C_{32} =$ *Unknown*
  end if
end for
Update Map_i

Identify patches of water among patches of $C_{22} =$ *Water OR Unknown*[(2)]
Create layer L with $P_{C_{31} \cup C_4}$ and layer L' with $P_{C_{22}}$
for all $(p_k^L, p_l^{L'}) \in L \times L'$ **do**
  if p_k^L and $p_l^{L'}$ adjacent **then**
    class$(p_l^{L'}) \leftarrow C_4$ # $C_4 =$ *Water*
  else
    class$(p_l^{L'}) \leftarrow C_{32}$ # $C_{32} =$ *Unknown*
  end if
end for
Update Map_i

Identify patches of dense forest among patches of class $C_6 =$ *Secondary growth OR Degraded forest OR Forest* [(3)]
Create layers L with $P_{C_7 \cup C_8 \cup C_9 \cup C_{10} \cup C_{11}}$ and layer L' with P_{C_6}
for all $(p_k^L, p_l^{L'}) \in L \times L'$ **do**
  if p_k^L and $p_l^{L'}$ adjacent **then**
    class$(p_l^{L'}) \leftarrow C_{61}$ # $C_{61} =$ *Secondary growth OR Degraded forest*
  else
    class$(p_l^{L'}) \leftarrow C_5$ # $C_5 =$ *Forest*
  end if
end for
Update Map_i

Finalize Map_i (expert-based):
Correct errors and label patches of $C_{32} =$ *Unknown* (with classes $C_5 =$ *Forest* and $C_{61} =$ *Secondary growth OR Degraded forest* essentially)
end for

Create the final land cover/use map, Map, by patching the maps Map_i:
$Map = \text{patch}(Map_1, Map_2, ..., Map_5)$

Finalize class interpretation and labeling and evaluate (expert knowledge, field works and BD-Ortho® aerial photographs):
Label classes as following: C_{11} = *No data (clouds)*; C_{21} = *No data (cloud shadows)*; C_{31} = *Bare soil OR buildings* ; C_4 = *Water*; C_5 = *Dense forest*; C_{61} = *Secondary OR Degraded forest* ; C_7 = *Monospecific and homogeneous vegetation*; C_8 = *Dense low vegetation with shrubs*; C_9 = *Dense low vegetation*; C_{10} = *Scattered herbaceous vegetation*; C_{11} = *Dry savannah*

(1) Human settlements are often nearby rivers in this amazonian region. Consequently, patches of C_3 = *Bare soil OR Building OR Unknown* nearby water are likely to correspond to bare soil or buildings. Small isolated patches of C_3 = *Bare soil OR Building OR Unknown* far from water often correspond to emergent trees with particular light colors.

(2) Patches initially considered as shadows and adjacent to water bodies are likely to correspond to shady and/or shallow water (river banks). Moreover, gold mining sites are characterized by patches of water, bare soil and patches initially considered as shadows that, in fact, correspond to shady and/or shallow water too.

(3) Patches of C_6 = *Secondary growth OR Degraded forest OR Forest* that are adjacent to patches of anthropogenic vegetation classes are likely to correspond to secondary growth or degraded forest and not to dense ”primary” forest.
